# Genome-wide siRNA screen of genes regulating the LPS-induced NF-κB and TNF-α responses in mouse macrophages

**DOI:** 10.1038/sdata.2017.8

**Published:** 2017-03-01

**Authors:** Ning Li, Samuel Katz, Bhaskar Dutta, Zachary L. Benet, Jing Sun, Iain D.C. Fraser

**Affiliations:** 1 Signaling Systems Unit, Laboratory of Systems Biology, Bethesda, Maryland 20892, USA; 2 Department of Veterinary Medicine, University of Cambridge, Madingley Road, Cambridge CB3 0ES, UK; 3 Bioinformatics Team, Laboratory of Systems Biology, National Institute of Allergy and Infectious Diseases, National Institutes of Health, Bethesda, Maryland 20892, USA

**Keywords:** Microarray analysis, Inhibitory RNA techniques, Toll-like receptors, High-throughput screening, Systems biology

## Abstract

The mammalian innate immune system senses many bacterial stimuli through the toll-like receptor (TLR) family. Activation of the TLR4 receptor by bacterial lipopolysaccharide (LPS) is the most widely studied TLR pathway due to its central role in host responses to gram-negative bacterial infection and its contribution to endotoxemia and sepsis. Here we describe a genome-wide siRNA screen to identify genes regulating the mouse macrophage TNF-α and NF-κB responses to LPS. We include a secondary validation screen conducted with six independent siRNAs per gene to facilitate removal of off-target screen hits. We also provide microarray data from the same LPS-treated macrophage cells to facilitate downstream data analysis. These data provide a resource for analyzing gene function in the predominant pathway driving inflammatory signaling and cytokine expression in mouse macrophages.

## Background & Summary

Toll-like receptors (TLR) are a major class of pattern recognition receptors that serve as the primary sensors of microbial stimuli in innate immune cells^1–3^. Due to their centrality in the host system’s response to microbial infection and the critical importance of the regulation and modulation of this response, cells with activated TLR receptors have been the target of genetic perturbation screens^4^, including several that have employed RNAi technology^5–8^. However, due to the challenges of efficient small RNA delivery as well as the non-specific immune response to dsRNA that can be induced by the application of the siRNA technology to innate immune cells^9–11^, genome-wide siRNA perturbation studies have broadly been limited to screens using fibroblast and mesenchymal cell lines or have used the recently developed CRISPR/Cas9 perturbation technology^12^. Given the immune specificity of its response and the complex known and unknown effects of various perturbation methods^13–15^, critical insights stand to be gained by studying the activation of TLR responses in hematopoietic differentiated cells under diverse genome-wide screening conditions. To address the absence of genome-wide siRNA screens in innate immune cells we set up a screen utilizing a screening platform we previously reported^16^, which uses a cloned mouse macrophage cell line containing dual assay readouts of NF-κB activation and a TNF-α transcriptional reporter, and stimulated these cells with lipopolysaccharide (LPS), a primary activator of the TLR4 receptor^1,17^. The TLR4 signaling pathway is the most heavily studied of the TLR family receptor pathways with an established central role in the host’s primary response to infection, and clinical significance in cases such as sepsis and endotoxemia^18,19^. Our screen provides a comprehensive genome-wide siRNA study of TLR4 response to LPS with dual readouts, demonstrating the effect of each gene’s perturbation by siRNA on two different downstream effectors of the inflammatory response; allowing for comprehensive, comparative, and dynamic analysis of the regulation of this critical pathway.

The screens used a library of siRNA SMARTpools, containing 4 siRNAs targeting 16,870 of the genes in the mouse genome. Two parallel screens were performed to enable different recording time points for the assay readouts of initial NF-κB activation and subsequent TNF-α transcriptional reporter activity. Following two days of transfection with siRNA, the cells in both sets of plates were stimulated with LPS. To correspond with the relevant time of observed peak activation, cells designated for the recording of NF-κB activation were fixed for imaging after 40 min incubation with LPS, while cells designated for the recording of TNF-α transcriptional reporter activity were fixed after 16 hours incubation with LPS. The two readouts were evaluated using high-content imaging analysis; the ratio of nuclear to cytosolic RelA translocation for NF-κB activation, and mCherry fluorescence intensity for the TNF-α transcriptional reporter. Wells with low cell numbers identified gene targets broadly effecting cell viability. Following standardization, novel hits from both assays were selected for a secondary screening assay where six individual siRNAs from two different vendors were used to facilitate removal of off-target hits from the primary screen. The readouts were analyzed as in the primary screen, with the results normalized to the relevant negative controls.

The two primary screening assays identified 717 positive and 44 negative regulators of NF-κB activation and 796 positive and 299 negative regulators of TNF-α induction. Bioinformatic analysis of these candidate genes prioritized 260 novel NF-κB regulators and 352 novel TNF-α regulators for secondary screening. The secondary screen identified 82 robust novel regulatory candidates for the LPS response in mouse macrophages, 64 of these gene targets showing effects on both the NF-κB and TNF-α readouts. Our screen thus provides a comprehensive dataset of putative regulators of the TLR4 response in innate immune cells, and can be used to validate and supplement results from similar studies done in other cell types, and also as a comparative analysis of studies using different perturbation technologies. The dual readouts from our reporter cells also provide a dynamic readout of putative regulators that, through more complex computational analysis, could illuminate the hierarchical regulatory architecture of the TLR4 signaling pathway.

## Methods

### Generation of RAW G9 reporter cell line for screening

**Figure 1:**
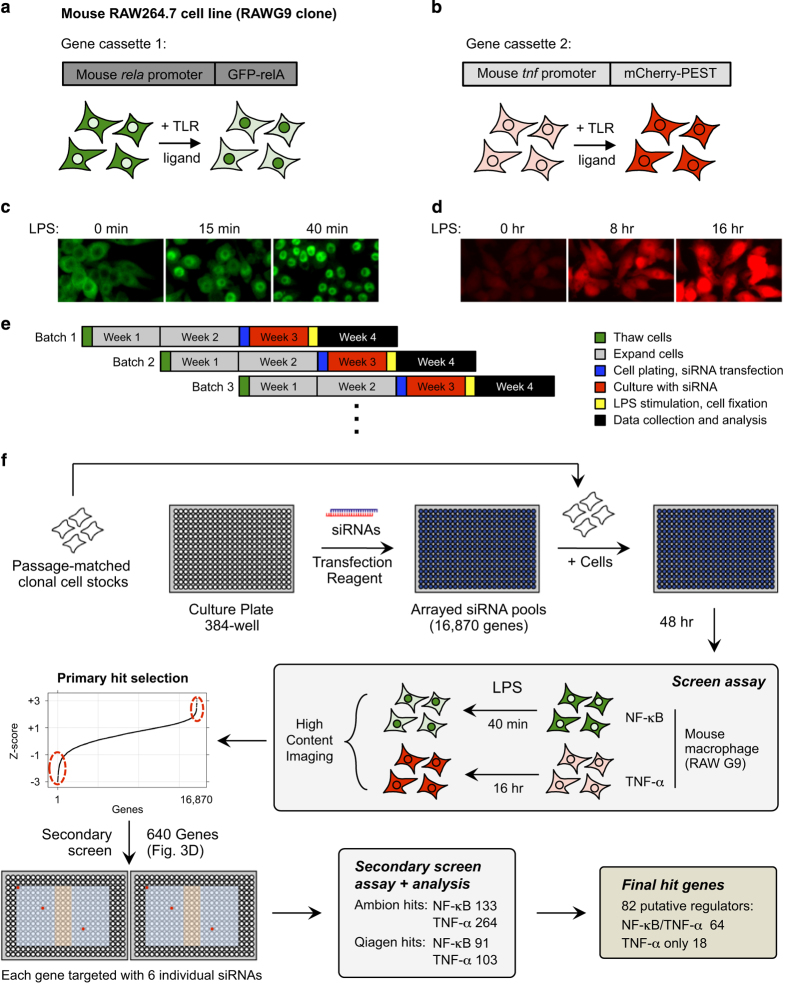
Overview of screen assay development and workflow. (a+b) Gene cassettes in the mouse RAW G9 reporter clone containing (**a**) the mouse *rela* promoter driving expression of a GFP-relA fusion protein and (**b**) the mouse *tnf* promoter driving expression of an mCherry-PEST fusion protein. (**c**) Cytosol-to-nuclear translocation of the GFP-relA fusion in RAW G9 cells up to 40 min after treatment with 10 ngml^−1^ LPS (**d**) Increased *tnf* promoter-driven mCherry expression in RAW G9 cells up to 16 h after treatment with 10 ngml^−1^ LPS at 16 h after treatment with LPS. (**a**-**d**) reproduced from Li *et al.*^16^ (**e**) Schematic of procedure to minimize cell line variation in screen. All RAW G9 cell batches used were from a single parental low passage stock. (**f**) Overview of the procedure for primary and secondary siRNA screens of the mouse macrophage NF-κB and TNF-α response to LPS.

While the generation of the RAW G9 reporter cells with dual readouts for NF-κB activation and TNF-α transcriptional reporter activity has been described previously^16,20^, we summarize the main features of the reporter cells again here. We constructed a lentiviral plasmid expressing dual gene cassettes for NF-κB and TNF-α assay reporters (Fig. 1a,b). The NF-κB reporter included the mouse *Rela* promoter driving a GFP fusion of the mouse *Rela* gene. The TNF-α reporter included the mouse *Tnf* promoter driving expression of the mCherry red fluorescent protein fused to a destabilizing PEST sequence to provide a dynamic readout for *Tnf* promoter activity. Lentiviral particles were generated and used to infect RAW264.7 cells as described previously^21^. Single cell clones were isolated and screened for optimal nuclear translocation of GFP-RelA and increase in mCherry fluorescence in response to LPS (Fig. 1c,d). To address the challenges of small RNA delivery to macrophages and non-specific innate responses to dsRNA, we targeted the stably expressed GFP reporter gene to develop efficient siRNA delivery protocols for maximal target gene silencing with minimal activation of the innate macrophage response to nucleic acids^16^.

### Cell culture and TLR ligand stimulation

RAW G9 cells were maintained in DMEM, 10%FBS, 20 mM Hepes, and 2 mM glutamine. A large batch of low passage RAW G9 cells sufficient for the entire screening process were prepared and frozen together. A new batch of cells were thawed each week throughout the screening process, and each batch were cultured for exactly 14 days prior to siRNA transfection, to ensure the same cell passage number was used for every experiment in the screen (Fig. 1e). LPS was from Alexis Biochemicals, Salmonella minnesota R595 TLRgrade, ALX-581-008-L002.

### High throughput siRNA screening

#### Overview

The genome-wide siRNA screens were run in 384-well format with duplicates of each siRNA plate prepared to measure the two different reporter readouts from the RAW G9 cells^16^: NF-κB activation measured by nuclear translocation of GFP-tagged RelA protein, and TNF-α transcriptional activation measured by murine *Tnf* promoter driven mCherry expression (Fig. 1f). We used the Dharmacon siGENOME siRNA mouse library, containing a single SMARTpool of 4 siRNAs targeting each of 16,870 genes across 55 plates. Replicate plates were run in successive weeks, and passage matched cells were used throughout the screening process to minimize cell line variability. Plates were prepared with siRNAs against target genes in columns 3-22, with controls (at least 3 wells each) in columns 1, 2, 23 and 24. siRNA concentration throughout the primary and secondary screens was fixed at 100 nM, previously identified as optimal for the RAW G9 cell line^16^. Negative controls included transfection lipid alone, non-targeting control (NTC) siRNA pools NTC2 and NTC5, and siRNA targeting the cyclophilin B gene (*Ppib*). Positive control target genes were chosen from the canonical TLR4 pathway across a range of expected phenotypic strength; *Tlr4, Myd88, Irak1* and *Ikbkg*. All cell plating and liquid handling steps were conducted with a Multidrop dispenser (Thermo Fisher) and EL406 plate washer/dispenser (Biotek). Plates were imaged on a BD Pathway 855 high content imager and images analysed with Attovision software.

### Screening reagents.

Dharmacon siGENOME siRNA mouse library (G-014650-02; G-013500-02; G-013600-02; G-015000-02).

Custom Ambion siRNA library (secondary screen).

Custom Qiagen siRNA library (secondary screen).

Negative control siRNAs (Dharmacon); NTC2 (D-001210-02), NTC5 (D-001210-05), *Ppib* (D-001136-01).

Positive control siRNAs; *Tlr4 (M-047487-01), Myd88 (M-06307-00), Ikbkg (M-040796-01), Irak1* (M-040116-01), custom siGFP siRNA^16^.

Imaging plates (BD Falcon, REF 353962).

Transit TKO transfection reagent (Mirus, MIR 2156).

Dulbecco’s phosphate buffered saline (DPBS) (Gibco, 14190-144).

DMEM (Lonza, Cat#: 12-614F).

Fetal bovine serum (GermCell, 100-500).

Hepes (Corning, 25-060-CL).

L-glutamine (Lonza, 17-605E).

LPS (Alexis Biochemicals, Cat# ALX-581-008-L002).

Hoechst 33342 (Invitrogen, H3570).

Paraformaldehyde, 16% solution (Electron Microscopy Sciences, 15710).

#### Day 1: siRNA transfection

All siRNAs were pre-arrayed in 384-well plates with 2 μl of a 2.5 μM master stock. To each well, 8 μl of DPBS containing 0.2 μl of Transit TKO was added and plates were shaken for 1 min to generate a homogenous siRNA/lipid suspension for the subsequent reverse transfection of cells. After 20 min of incubation at room temperature, a 40 μl suspension of 5,000 cells in growth media was added to give a final siRNA concentration of 100 nM. Cells were incubated at 37 °C/5% CO_2_ for 48 h.

#### Day 3: LPS stimulation and NF-κB reporter imaging data collection

The media on the cells was changed to 40 μl fresh complete growth medium containing 10 ngml^−1^ LPS, apart from control wells run with no LPS stimulation, which received media alone. At this stage, Hoechst nuclear stain was also added to all wells to a final concentration of 0.6 μgml^−1^. After 40 min of incubation, cells in the NF-κB readout plates were fixed with 4% paraformaldehyde for 10 min, washed, and then maintained in DPBS until imaging. Incubation was continued overnight for the cells in the TNF-α readout plate.

#### Day 4: TNF-α reporter imaging data collection

After 16 h of incubation with 10 ngml^−1^ LPS, cells in the TNF-α readout plates were fixed with 4% paraformaldehyde for 10 min, washed, and then maintained in DPBS until imaging.

#### Image analysis

The NF-κB and TNF-α readout plates were imaged using a BD Pathway 855 bioimager (BD biosciences). Two imaging fields were collected from each well with a 20×objective using Laser Autofocus, providing imaging data for approximately 300-400 cells per well. Exposure times were as follows: Hoechst nuclear stain=0.25 s, GFP channel=0.2 s, mCherry channel=0.3 s. BD AttoVision software was used to automatically identify and quantify Hoechst-stained cell nuclei, GFP-p65 fluorescence and mCherry fluorescence. For both the GFP and mCherry channels, background signal was calculated from regions of the imaging field between cells and was automatically subtracted from the reporter signal using the BD AttoVision software. GFP signal intensity located within the area of the nuclear stain (eroded by 2 pixels) was defined as nuclear NF-κB, while GFP within a 2-pixel-wide ring outside the nuclear staining was defined as cytosolic NF-κB. The 2-pixel cytosolic ring was set 1 pixel outside of the nuclear region. For determination of NF-κB translocation, the ratio of nuclear to cytoplasmic GFP-p65 intensity was calculated using BD Image Data Explorer software. For mCherry expression, nuclear mCherry was quantified using the same method as for NF-κB, and the average mCherry intensity was used as a measure of TNF-α promoter activity. Cell number was also recorded from each well imaging field as a measure of cell viability.

#### Data analysis

**Figure 2:**
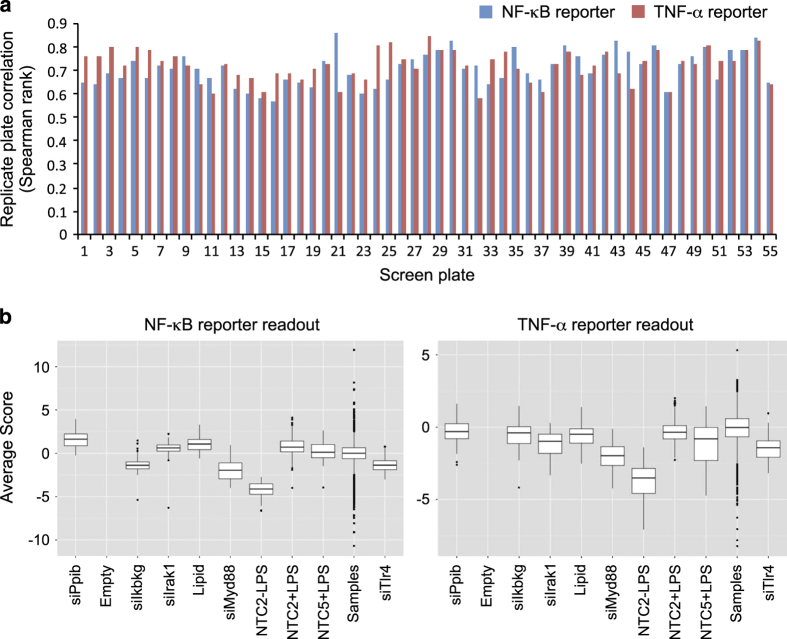
Primary screen QC metrics. (**a**) Replicate correlation across all 55 primary screen plates for both the NF-κB and TNF-α screen reporter readouts. (**b**) Boxplots of negative and positive control performance in the primary screens for both the NF-κB and TNF-α screen reporter readouts.

For the primary siRNA screen, data was first normalized on a per-plate basis to the intra-plate median. We then standardized the values for each replicate experiment using the robust z-score calculation^22^. We evaluated the correlation between replicate plates (Fig. 2a), and set a minimal correlation (Spearman rank) of 0.55. If plates did not meet this reproducibility threshold, plates were repeated until higher correlation was observed.

### siRNA screen hit selection

#### Primary genome-wide screen.

##### Analysis

**Figure 3:**
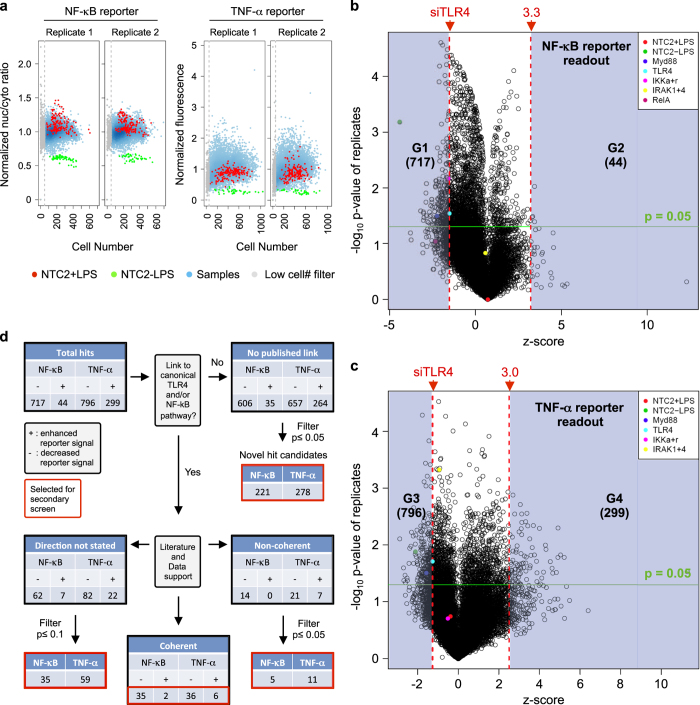
Primary screen analysis and hit selection. (**a**) Dot plots showing cell numbers per well for the NF-κB readout (nuclear/cytosolic ratio) and the TNF-α reporter (mCherry fluorescence) in the primary screen. A cutoff of 50 cells was used to filter out wells where target gene knockdown led to a cell growth phenotype. (**b** and **c**) Volcano plots of replicate -log *p*-value against screen score for genes in the (**b**) NF-κB and (**c**) TNF-α readout screens. Red dashed lines show hit thresholds selected and green line shows p-value cutoff of 0.05. Gene numbers selected in groups G1 to G4 are shown and were used as inputs for analysis workflow. (**d**) Informatics analysis workflow for genes in G1-G4 groups selected from (**c**,**d**). Genes highlighted in red boxes were selected for the secondary screen.

Upon completion of the primary screen, we first evaluated the average cell number per field and set a minimum threshold of 50 cells per well to remove genes whose knockdown had a substantial effect on cell viability in both screen replicates (Fig. 3a, Data records 1 and 2; LowCellCount field). The primary screen data distribution histograms showed normally distributed data for both readouts. Boxplots of the screen samples against controls are shown in Fig. 2b. The mean of the robust z-score from replicate samples was taken as the final score for each gene. We also performed a 2-sided students t-test on the replicate data for each gene to generate an associated *p*-value for each gene, and volcano plots of screen scores versus −log *P*-value are shown for NF-κB (Fig. 3b) and TNF-α (Fig. 3c). We chose the median score for the TLR4 siRNA control (−1.56 for NF-κB and −1.3 for TNF-α) as the threshold for putative positive regulators of the LPS-induced NF-κB and TNF-α activation. Robust z-scores of ≥3.3 and ≥3.0 were chosen for putative negative regulators of the NF-κB and TNF-α readouts respectively. This initially identified a set of 717 positive (Fig. 3b, G1 group), and 44 negative (Fig. 3b, G2 group) regulators of NF-κB, and 796 positive (Fig. 3c, G3 group), and 299 negative (Fig. 3c, G4 group) regulators of TNF-α. These genes were then subjected to an informatics analysis workflow using Ingenuity Pathway Analysis (IPA) software which considered their known links to the TLR4 pathway and/or NF-κB activation, along with the *p*-value calculated from their replicate robust z-scores (Fig. 3d). This identified a putative hit list of 688 genes (Fig. 3d, red boxes). This group included 48 genes identified from the canonical TLR4 pathway, but these genes were not selected for the secondary screen (see ‘Identification of known pathway regulators’ section under Technical Validation), as our goal was to identify novel regulators of the LPS response. This analysis resulted in a selected gene list of 260 NF-κB regulators, 352 TNF-α regulators and 28 genes regulating both assay readouts, for a total primary screen putative hit list of 640 genes.

#### Secondary screen with six independent siRNAs per gene.

##### Methodology

It has been shown that siRNA screens are subject to a significant frequency of off-target effects (OTEs) driven by the seed sequence of siRNAs targeting the 3′UTR of unintended gene targets by a microRNA-like targeting mechanism^13,14^. The most reliable method to separate off-target from on-target hits in an siRNA screen is to target putative hits with alternative siRNA sequences (containing different seeds) in the secondary screen. We therefore employed an additional six siRNAs from alternate vendors (three each from Ambion and Qiagen) for each of the hit genes from the primary screen. While three siRNAs for all 640 genes were available from Qiagen, Ambion did not have available siRNAs for 27 genes, so an additional 27 putative negative regulators of the TNF-α response were selected from the primary screen analysis to complete a total secondary screen gene set of 667 genes (siRNA for 640 genes from each vendor, 613 common, 27 Qiagen only, 27 Ambion only). The Gene Symbols and Entrez IDs for the 667 genes are included in Data records 3 and 4. The secondary screen siRNAs were plated in 384-well format with the outer rows A, C, O and P and columns 1, 2, 22, 23 and 24 left empty and three central columns (11, 12 and 13) left open for control siRNAs (see secondary screen plate map in Fig. 1f, workflow). Negative and positive controls were the same as used for the primary screen. Also, individual siRNAs for each gene were plated in separate regions of the plate (Fig. 1f, wells highlighted red show example for one gene). The secondary screen was run in 384-well format with duplicates of each siRNA plate prepared to measure the two different reporter readouts from the RAW G9 cells^16^; NF-κB activation at 40 min was again measured by nuclear translocation of GFP-tagged RelA protein, and TNF-α transcriptional activation at 16 h was measured by murine *Tnf* promoter driven mCherry expression (see Fig. 1f, Screen assay). Replicate plates were run in successive weeks, and passage matched cells (from the same parental cell stock as the primary screen) were again used to minimize cell line variability (Fig. 1e).

##### Analysis

**Figure 4:**
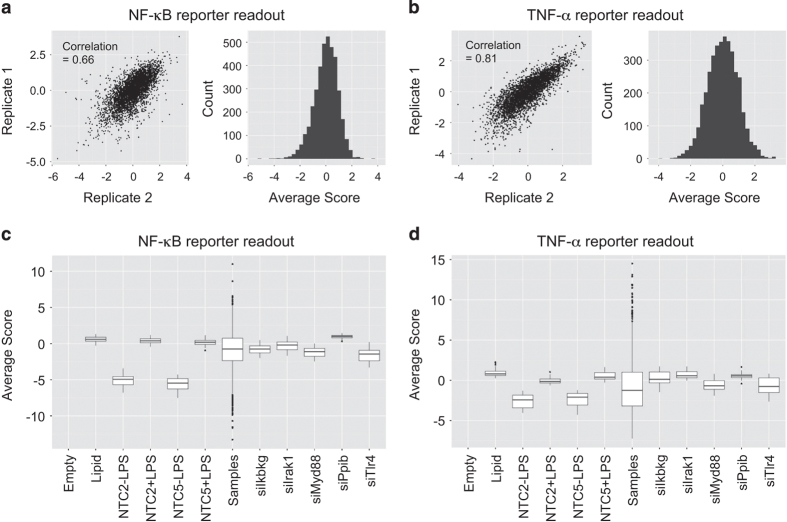
Secondary screen QC metrics. (**a** and **b**) Replicate correlations and data distribution histograms for the (**a**) NF-κB and (**b**) TNF-α reporter readouts in the secondary LPS screens. (**c** and **d**) Boxplots of negative and positive control performance in the secondary screens for the (**c**) NF-κB and (**d**) TNF-α screen reporter readouts.

For the secondary siRNA screen, data was again normalized on a per-plate basis to the intra-plate median, however robust z-score was less satisfactory for hit selection due to the higher frequency of putative hits among the screened genes. Secondary screen z scores were therefore generated using a ‘fraction of negative control’ calculation using values from the NTC control siRNAs^22^. We focused our secondary screen analysis on the 613 genes for which we had six independent siRNAs, three each from Ambion and Qiagen. We calculated z-scores from two replicate secondary screen experiments, and observed satisfactory correlations of >0.6 for both readouts (Fig. 4a,b). We then used transcription profiling data to add present/absent expression calls (see Transcriptome analysis), which selected 366 secondary screen genes with clear expression in mouse macrophages for further analysis. We then calculated median z-scores separately for the siRNAs from each vendor (median taken from 6 scores per gene; 3 siRNA×2 replicates), to obtain four scores for each gene; Ambion TNF-α and NF-κB readouts and Qiagen TNF-α and NF-κB readouts (Supplementary Table 1). We observed the phenotypic strength of the scores to be weaker for the Qiagen siRNA set than the Ambion set, with consistently lower median z-scores for the Ambion siRNAs for both readouts (Ambion TNF-α −2.89, NF-κB −1.50; Qiagen TNF-α −0.07, NF-κB −0.04), and median z-score differences per gene of −2.69 for TNF-α and −1.37 for NF-κB. We therefore chose to set a hit threshold for positive regulators of the LPS response at −2 for the Ambion scores and −1 for Qiagen. This classified the following gene numbers as validated positive regulator hits: Ambion TNF-α 264, Ambion NFκB 133, Qiagen TNF-α 103 and Qiagen NFκB 91 (Supplementary Table 1). We then ranked genes with the highest representation in these four groups (SumAllhit), finding 20 genes that were hits in all 4 groups, 44 genes present in 3 of 4, and then 18 genes scoring positive in both TNF-α groups. We did not find any genes that gave a strong NF-κB phenotype with no effect on TNF-α, consistent with the important role for this transcription factor in expression of the mouse TNF-α gene. We also found relatively few genes with consistent negative regulatory effects on the pathway with siRNAs from both vendors. We therefore identified 82 novel candidate positive regulators of the mouse LPS response (Supplementary Table 1), 64 of these showed effects on both NF-κB activation and TNF-α induction, while a further 18 had more direct effects on TNF-α.

### Transcriptome analysis

RAW G9 cells were cultured as described in the ‘Cell culture and TLR ligand stimulation’ section above and were prepared at the same passage number as cells used for siRNA screening. The media on the cells was changed to fresh complete growth medium containing 10 ngml^−1^ LPS, apart from control wells run with no LPS stimulation, which received media alone. Cells were incubated for 4 h and total RNA was isolated from approximately 10^6^ cells per condition using an RNAeasy Mini Kit (Qiagen). Each condition was represented by biological duplicates. Complementary RNA amplification and labeling were performed using the Illumina TotalPrep RNA Amplification Kit (Ambion), microarray hybridization and scanning protocols followed standard Illumina protocols. Signal data was extracted from the image files with the Gene Expression module (v.1.9.0) of the GenomeStudio software (v.2011.1), and Log_2_ signal intensity and detection *p*-values were determined. Genes with *p*-value of detection of less than 0.1 were considered expressed. The same method of expression detection was determined for genes in mouse primary macrophages using a previously published microarray dataset^20^. Genes were considered expressed in macrophages if they had a *p*-value of detection of less than 0.1 in at least one of the 4 conditions analyzed (+/−LPS in RAW G9 cells or primary macrophages).

## Data Records

### Data record 1

Primary screen siRNA data for the NF-κB reporter readout are available at PubChem under the accession number AID 1224828 (Data Citation 1). Raw data for the two replicate experiments for NF-κB/RelA nuclear:cytoplasmic ratio (Rep1-2Value), and the cell count per well (Rep1-2CellCount) are provided. Normalized data for the NF-κB reporter is also included (Zscore), filtered wells with low cell count and control wells are indicated, and samples are defined by siRNA SMARTpool ID (Dharmacon catalog number), Gene Symbol and EntrezID. Minimal data fields for analysis of the screen data using CARD software^23^ are included (PlateID, Well, GeneSymbol, EntrezID, siRNAID, WellAnno). The PubChem activity score indicates the phenotypic outcome for each well (0= Z-score between 1 and −1; 25= Z-score greater than 1 or less than −1; 50= Z-score greater than 3.3 or less than −1.56; 75= Z-score greater than 4 or less than −2; 100= Z-score greater than 5 or less than −2.5), and the Pubchem activity outcome notes whether the siRNA SMARTpool in a given well was considered ‘active’=2 or ‘inactive’=1. Note that all control wells were assigned a 0 activity score and an outcome of 4 by default.

### Data record 2

Primary screen siRNA data for the TNF-α reporter readout are available at PubChem under the accession number AID 1224826 (Data Citation 2). Raw data for the two replicate experiments for TNF-α promoter driven mCherry fluorescence (Rep1-2Value), and the cell count per well (Rep1-2CellCount) are provided. Normalized data for the TNF-α reporter is also included (Zscore), filtered wells with low cell count and control wells are indicated, and samples are defined by siRNA SMARTpool ID (Dharmacon catalog number), Gene Symbol and EntrezID. Minimal data fields for analysis of the screen data using CARD software^23^ are included (PlateID, Well, GeneSymbol, EntrezID, siRNAID, WellAnno). The PubChem activity score indicates the phenotypic outcome for each well (0= Z-score between 1 and −1; 25= Z-score greater than 1 or less than −1; 50= Z-score greater than 3 or less than −1.3; 75= Z-score greater than 3.5 or less than −1.5; 100= Z-score greater than 4 or less than −2), and the Pubchem activity outcome notes whether the siRNA SMARTpool in a given well was considered ‘active’=2 or ‘inactive’=1. Note that all control wells were assigned a 0 activity score and an outcome of 4 by default.

### Data Record 3

Secondary screen siRNA data for the NF-κB reporter readout are available at PubChem under the accession number AID 1224829 (Data Citation 3). Raw data for the two replicate experiments for NF-κB/RelA nuclear:cytoplasmic ratio (Rep1-2Value) are provided. Normalized data is also included (ZscoreRep1-2), along with the replicate average for each individual siRNA (ZscoreAv) and then the median value for the three vendor-specific siRNAs targeting the same gene (ZscoreGeneMedian; separate values for Ambion and Qiagen, see Secondary screen Analysis section). Control wells are indicated, and samples are defined by siRNA ID (Ambion and Qiagen catalog numbers), siRNA# (1–6 for each gene target), Gene Symbol and EntrezID. Minimal data fields for analysis of the screen data using CARD software^23^ are included (PlateID, Well, GeneSymbol, EntrezID, siRNAID, WellAnno). The PubChem activity score indicates the phenotypic outcome for each well (0= Z-score between 1 and −1; 25= Z-score greater than 1 or less than −1; 50= Z-score greater than 2 or less than −2; 75= Z-score greater than 3 or less than −3; 100= Z-score greater than 4 or less than −4), and the Pubchem activity outcome notes whether the single siRNA in a given well was considered ‘active’=2 or ‘inactive’=1. Note that all control wells were assigned a 0 activity score and an outcome of 4 by default.

### Data record 4

Secondary screen siRNA data for the TNF-α reporter readout are available at PubChem under the accession number AID 1224827 (Data Citation 4). Raw data for the two replicate experiments for TNF-α promoter driven mCherry fluorescence (Rep1-2Value) are provided. Normalized data is also included (ZscoreRep1-2), along with the replicate average for each individual siRNA (ZscoreAv) and then the median value for the three vendor-specific siRNAs targeting the same gene (ZscoreGeneMedian; separate values for Ambion and Qiagen, see Secondary screen Analysis section). Control wells are indicated, and samples are defined by siRNA ID (Ambion and Qiagen catalog numbers), siRNA# (1–6 for each gene target), Gene Symbol and EntrezID. Minimal data fields for analysis of the screen data using CARD software^23^ are included (PlateID, Well, GeneSymbol, EntrezID, siRNAID, WellAnno). The PubChem activity score indicates the phenotypic outcome for each well (0= Z-score between 1 and −1; 25= Z-score greater than 1 or less than −1; 50= Z-score greater than 2 or less than −2; 75= Z-score greater than 3 or less than −3; 100= Z-score greater than 4 or less than −4), and the Pubchem activity outcome notes whether the single siRNA in a given well was considered ‘active’=2 or ‘inactive’=1. Note that all control wells were assigned a 0 activity score and an outcome of 4 by default.

### Data record 5

Microarray data from LPS treated and untreated RAW264.7 cells are available at the Gene Expression Omnibus (GEO) within the data series record GSE83826 (Data Citation 5). Two replicate data files are provided for each condition, containing Log_2_ signal intensity and detection *p*-values for each Illumina probe ID.

## Technical Validation

### Plate uniformity

Upon establishing the dual reporter screening assay in RAW G9 macrophages, we initially assessed the plate uniformity of the assay in 384-well format to determine if we could use the entire plate and avoid edge effects and other positional biases. Following the guidelines established by the NCATS screening facility^24^, we ran plate replicates over multiple days with different doses of LPS that gave increasing levels of activation of the screen reporters. This permitted testing of intra-plate uniformity within plates run on the same day and inter-plate variation across plates run on separate days. We confirmed that both NF-κB and TNF-α reporter assays met the recommended criteria of low intra-plate variation of CV <20% for different doses of LPS (Table 1). We also observed low inter-plate variation where the normalized mid-level signal maintain a fold shift of <2 between plates run across separate days (0.097 fold shift for NF-κB, 0.105 fold shift for TNF-α).

### Replicate correlation

Replicate correlation was evaluated throughout both the primary and secondary screens. Replicate plates were run in successive weeks, and any plates failing to achieve replicate correlation >0.55 were repeated. Replicate correlations for both assay readouts across the 55 plates in the primary screen are shown in Fig. 2a, and for the NF-κB and TNF-α secondary screens in Fig. 4a,b.

### siRNA transfection efficiency

In developing the RAW G9 reporter clone, we established a method for optimizing siRNA transfection efficiency whereby we targeted the constitutively expressed GFP-RelA reporter with a pool of potent siRNAs against GFP^16^. We included 4 siGFP and 4 NTC2 control wells on every screening plate that were not treated with LPS and were imaged in the GFP channel to assess siRNA transfection efficiency by reduction of the GFP signal. Average GFP knockdown levels were >80% throughout the primary and secondary screens (Table 2).

### Control performance

We included multiple positive controls on every screening plate targeting known components in the LPS/TLR4 pathway; the TLR4 receptor, the Myd88 signalling adapter, the proximal TLR pathway kinase IRAK1, and the Ikbkg component of the NF-κB pathway IKK kinase complex. Boxplots from the primary (Fig. 2b) and secondary screens (Fig. 4c,d), show varying levels of pathway perturbation with these controls, which we used as a metric for assessing thresholds for likely hits in the screen.

### Identification of known pathway regulators

The primary genome-wide screen identified numerous known components of the TLR4 signaling pathway for both screen readouts. Among the NF-κB reporter screen top hits were the signaling components *Tlr4* (−2.38), *Myd88* (−2.66), *Peli1* (−1.91), *Rela* (−2.42) and *Chuk* (−1.75), while the TNF-α reporter screen also identified *Myd88* (−2.01), *Tlr4* (−1.31) and *Peli1* (−1.3), in addition to the TNF-α inducing transcription factors *Elf4* (−2.24), *Spi1* (−1.55) and *Elk1* (−1.38). We chose not to include canonical pathway regulators in the secondary screen described here, as we have previously published a rigorous RNAi screen of over 100 genes from the canonical TLR pathways, using multiple siRNAs per gene and stimulating macrophages with a panel of up to six TLR ligands^8^.

## Usage Notes

### Data files for screen analysis using CARD software

We recently described a software package for comprehensive analysis of RNAi screen data, which combines both existing and novel algorithms for data pre-processing, reducing false positive hits through gene expression and off-target filtering, implementing network/pathway enrichment of high-confidence hits and predicting active miRNAs^23^. The data we describe in Data records 1 through 4 (Data Citations 1 through Data Citations 4) include all the required fields to permit analysis of the screen data in CARD (PlateID, Well, GeneSymbol, EntrezID, siRNAID, WellAnno). Instructions for uploading and analyzing the data in CARD have been previously described^23^.

## Additional Information

**How to cite**: Li, N. *et al.* Genome-wide siRNA screen of genes regulating the LPS-induced NF-κB and TNF-α responses in mouse macrophages. *Sci. Data* 4:170008 doi: 10.1038/sdata.2017.8 (2017).

**Publisher’s note**: Springer Nature remains neutral with regard to jurisdictional claims in published maps and institutional affiliations.

## Supplementary Material



Supplementary Information

## Figures and Tables

**Table 1:** Assessment of the plate uniformity of the NF-κB and TNF-α screening assays (see Technical Validation section).

	**High dose (LPS: 10 ngml^−1^)**	**Middle dose (LPS: 1 ngml^−1^)**	**Low dose (LPS: 0 ngml^−1^)**
Plate set 1 variation	NF-κB: 4.3%TNF-α: 8.6%	NF-κB: 4.4%TNF-α: 7.0%	NF-κB: 5.2%TNF-α: 7.6%
Plate set 2 variation	NF-κB: 3.9%TNF-α: 8.1%	NF-κB: 4.2%TNF-α: 7.0%	NF-κB: 4.4%TNF-α: 5.9%
Plate set 3 variation	NF-κB: 4.2%TNF-α: 7.3%	NF-κB: 3.8%TNF-α: 6.9%	NF-κB: 4.7%TNF-α: 6.8%

**Table 2:** Knock down efficiency of GFP fluorescence by GFP control siRNA in primary and secondary screens.

	**Mean**	**SD**
Primary screen	81.3%	0.63%
Secondary screen	84.1%	0.59%
